# Supplementary material for: Stress Responses and Ammonia Nitrogen Removal Efficiency of Oocystis lacustris in Saline Ammonium-Contaminated Wastewater Treatment
Source: Toxics. 2024 May 10;12(5):353. doi: 10.3390/toxics12050353 (PMC11125631; doi:10.3390/toxics12050353)
Supplement: Supplementary file 1 [file toxics-12-00353-s001.zip › toxics-2951887-supplementary.pdf]

Supplementary Material for

Stress Responses and Ammonia Nitrogen Removal Efficiency of *Oocystis lacustris* in Saline Ammonium-Contaminated Wastewater Treatment

Yuqi Zhu [1], Yili Zhang [1], Hui Chen [2], Lisha Zhang [1] and Chensi Shen [1,*]

[1] College of Environmental Science and Engineering, Donghua University, Shanghai 201620, China; 18017207003@163.com (Y.Z.); 17792797372@163.com (Y.Z.); lszhang@dhu.edu.cn (L.Z.)

[2] Key Laboratory of Agricultural Germplasm Resources Mining and Environmental Regulation of Ningbo City, College of Science and Technology, Ningbo University, Cixi 315302, China; chenhui07@126.com

* Correspondence: shencs@dhu.edu.cn

Figures

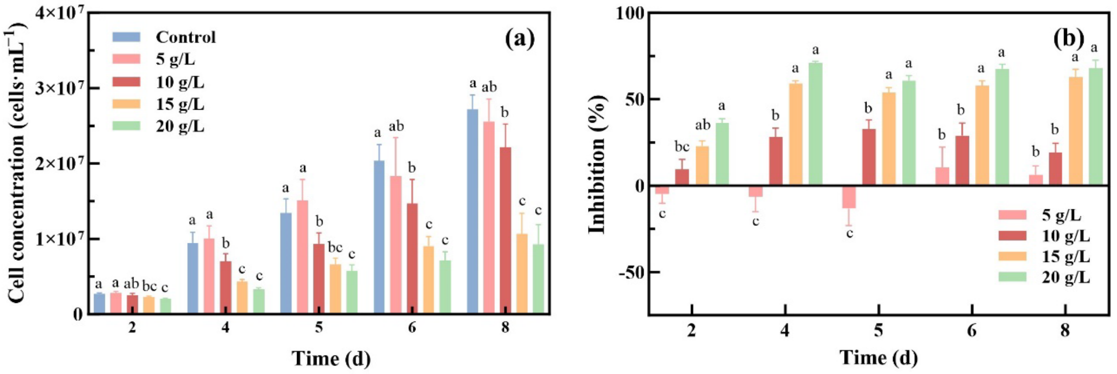

Figure S1. Growth of *Oocystis lacustris* at different Na_2SO_4 concentrations - September 2022. (**a**) Cell concentration; (**b**) inhibition of yield. (At the same cultivation time, different letters on adjacent bars indicate significant differences ($p < 0.05$), while the same letter indicates no significant difference.)

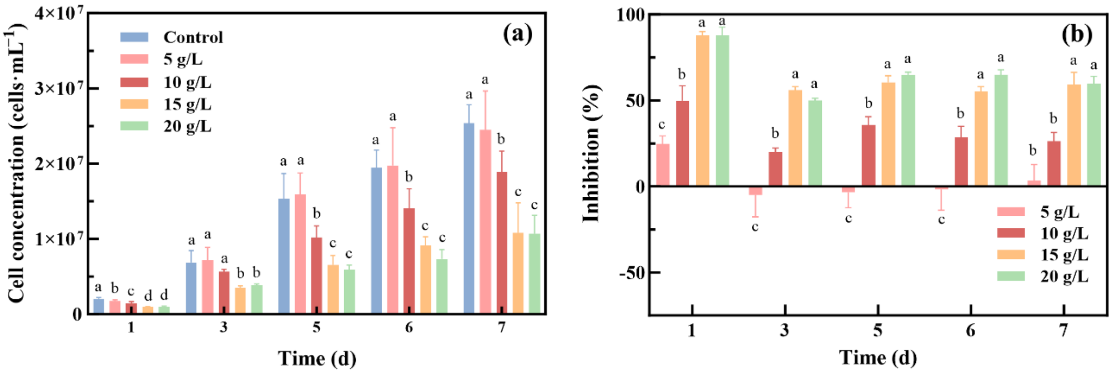

Figure S2. Growth of *Oocystis lacustris* at different Na_2SO_4 concentrations - November 2022. (**a**) Cell concentration; (**b**) inhibition of yield. (At the same cultivation time, different letters on adjacent bars indicate significant differences ($p < 0.05$), while the same letter indicates no significant difference.)

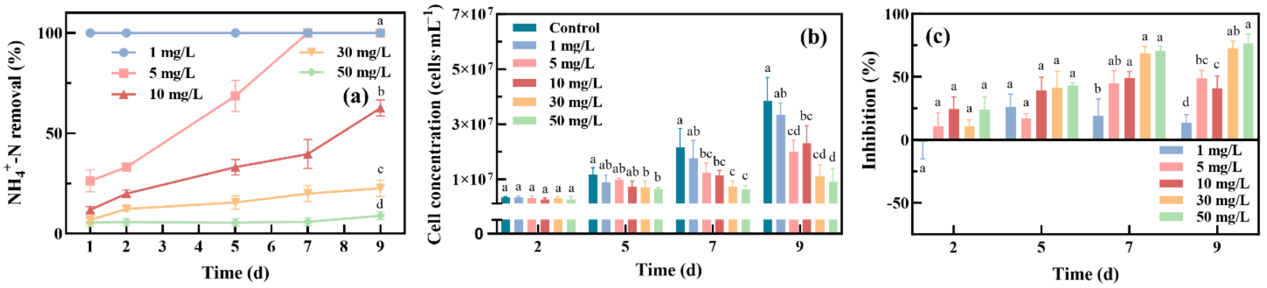

Figure S3. Treatment of *Oocystis lacustris* in the EX phase (8×10^5 cells/mL) with different concentrations of NH_4^+-N - September 2022. (**a**) NH_4^+-N removal rate; (**b**) cell concentration; (**c**) inhibition of yield. (At the same cultivation time, different letters on adjacent bars indicate significant differences ($p < 0.05$), while the same letter indicates no significant difference.)

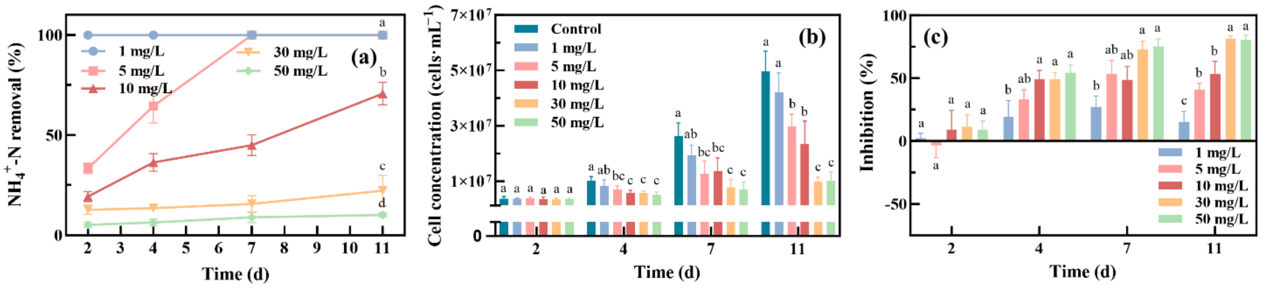

Figure S4. Treatment of *Oocystis lacustris* in the EX phase (8×10^5 cells/mL) with different concentrations of NH_4^+-N - November 2022. (**a**) NH_4^+-N removal rate; (**b**) cell concentration; (**c**) inhibition of yield. (At the same cultivation time, different letters on adjacent bars indicate significant differences ($p < 0.05$), while the same letter indicates no significant difference.)

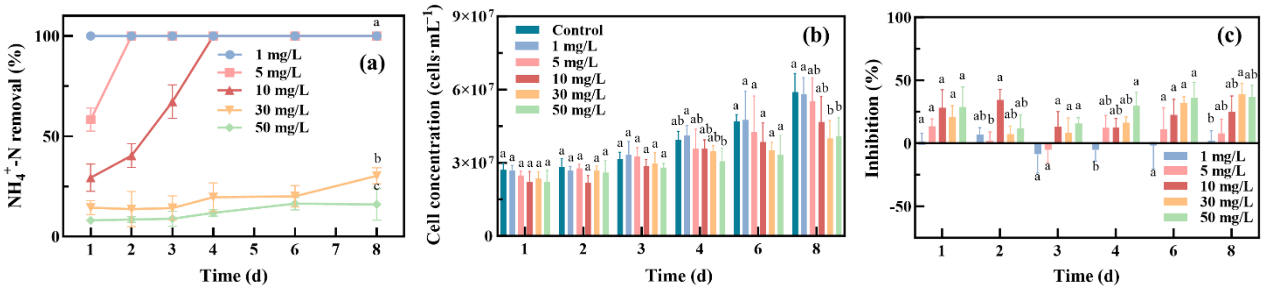

Figure S5. Treatment of *Oocystis lacustris* in the STA phase (2 × 10^7 cells/mL) with different concentrations of NH_4^+-N - September 2022. (**a**) NH_4^+-N removal rate; (**b**) cell concentration; (**c**) inhibition of yield. (At the same cultivation time, different letters on adjacent bars indicate significant differences ($p < 0.05$), while the same letter indicates no significant difference.)

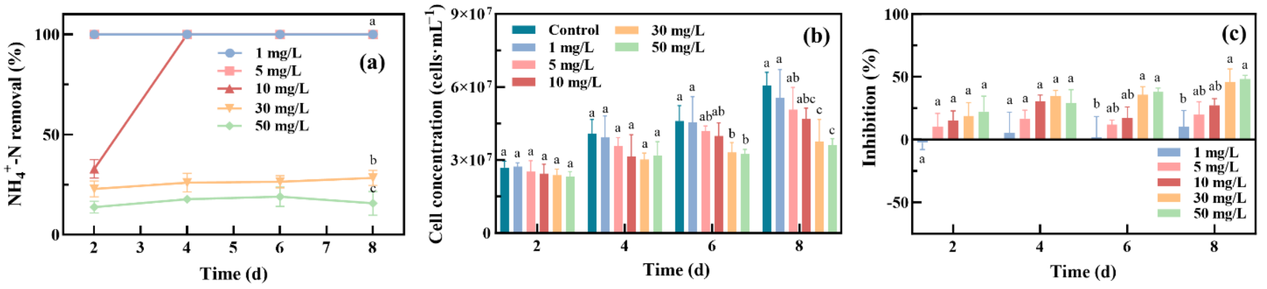

Figure S6. Treatment of *Oocystis lacustris* in the STA phase (2 × 10^7 cells/mL) with different concentrations of NH_4^+-N – November 2022. (**a**) NH_4^+-N removal rate; (**b**) cell concentration; (**c**) inhibition of yield. (At the same cultivation time, different letters on adjacent bars indicate significant differences ($p < 0.05$), while the same letter indicates no significant difference.)

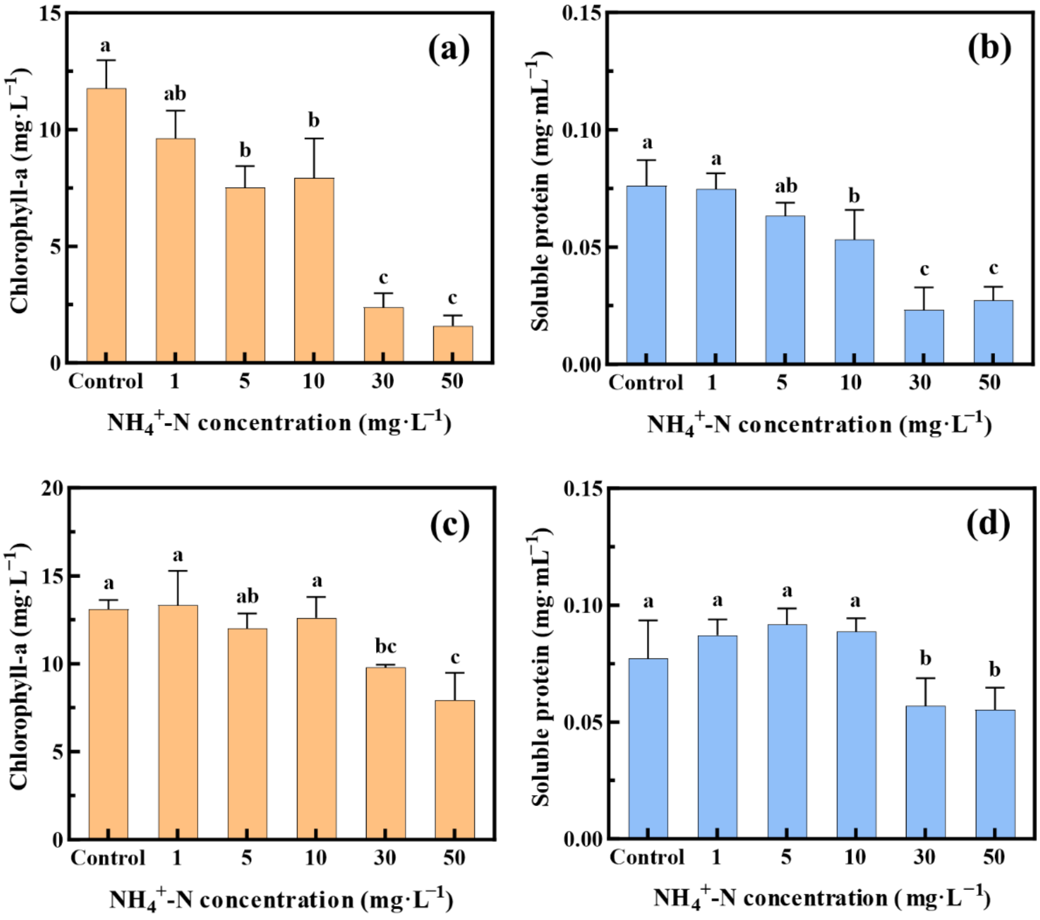

Figure S7. Variations in chlorophyll-a and soluble protein contents within *Oocystis lacustris* cells at different NH_4^+-N concentrations on the 8th day - September 2022. (**a,b**) Changes in chlorophyll-a and soluble protein contents within algal cells in the EX phase; (**c,d**) changes in chlorophyll-a and soluble protein contents within algal cells in the STA phase. (Different letters on adjacent bars indicate significant differences ($p < 0.05$), while the same letter indicates no significant difference.)

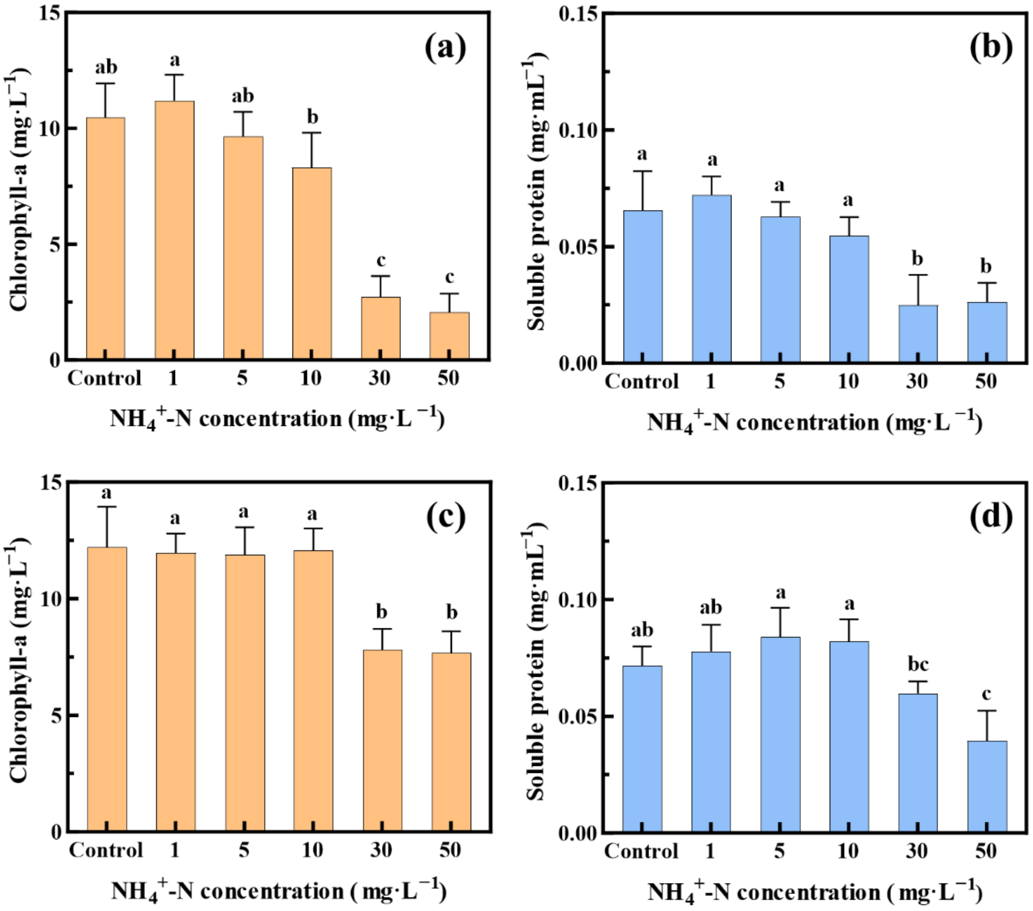

Figure S8. Variations in chlorophyll-a and soluble protein contents within *Oocystis lacustris* cells at different NH_4^+-N concentrations on the 8th day - November 2022. (**a,b**) Changes in chlorophyll-a and soluble protein contents within algal cells in the EX phase; (**c,d**) changes in chlorophyll-a and soluble protein contents within algal cells in the STA phase. (Different letters on adjacent bars indicate significant differences ($p < 0.05$), while the same letter indicates no significant difference.)

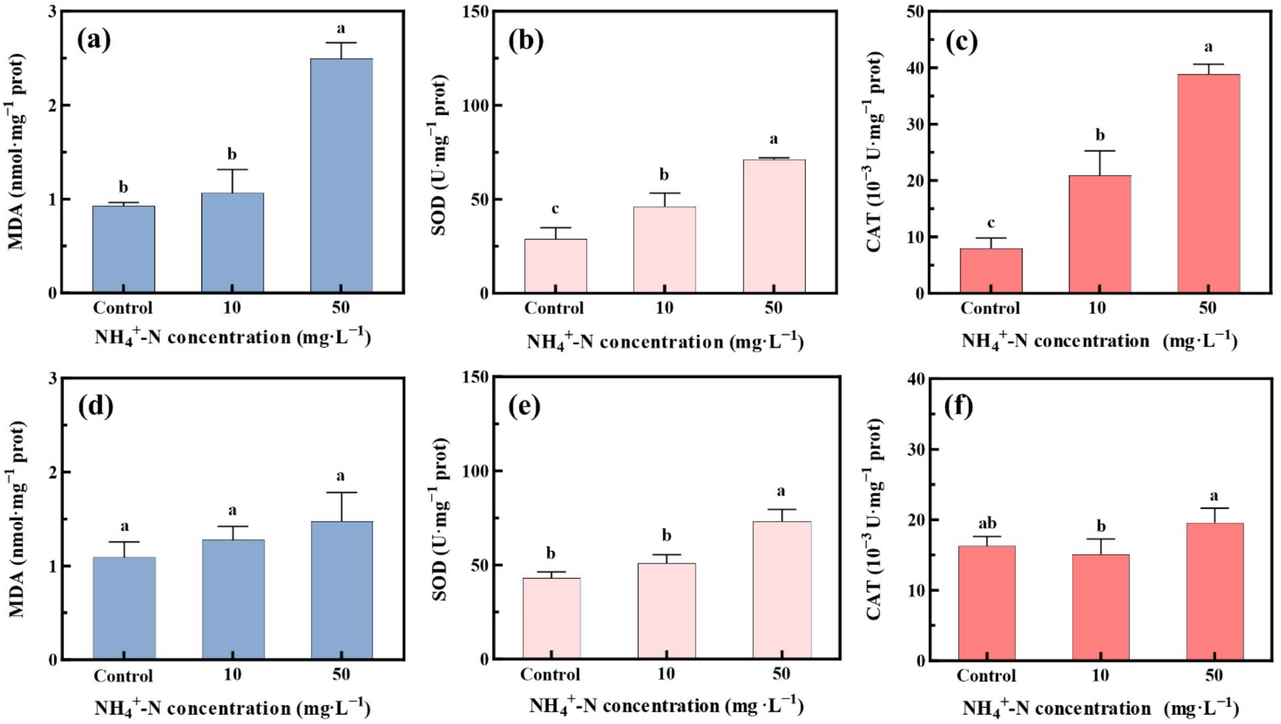

Figure S9. Oxidative stress status within *Oocystis lacustris* cells treated with different concentrations of NH_4^+-N on the 8th day - September 2022. (**a–c**) MDA concentration and SOD and CAT activities within algal cells in the EX phase; (**d–f**) MDA concentration and SOD and CAT activities within algal cells in the STA phase. (Different letters on adjacent bars indicate significant differences ($p < 0.05$), while the same letter indicates no significant difference.)

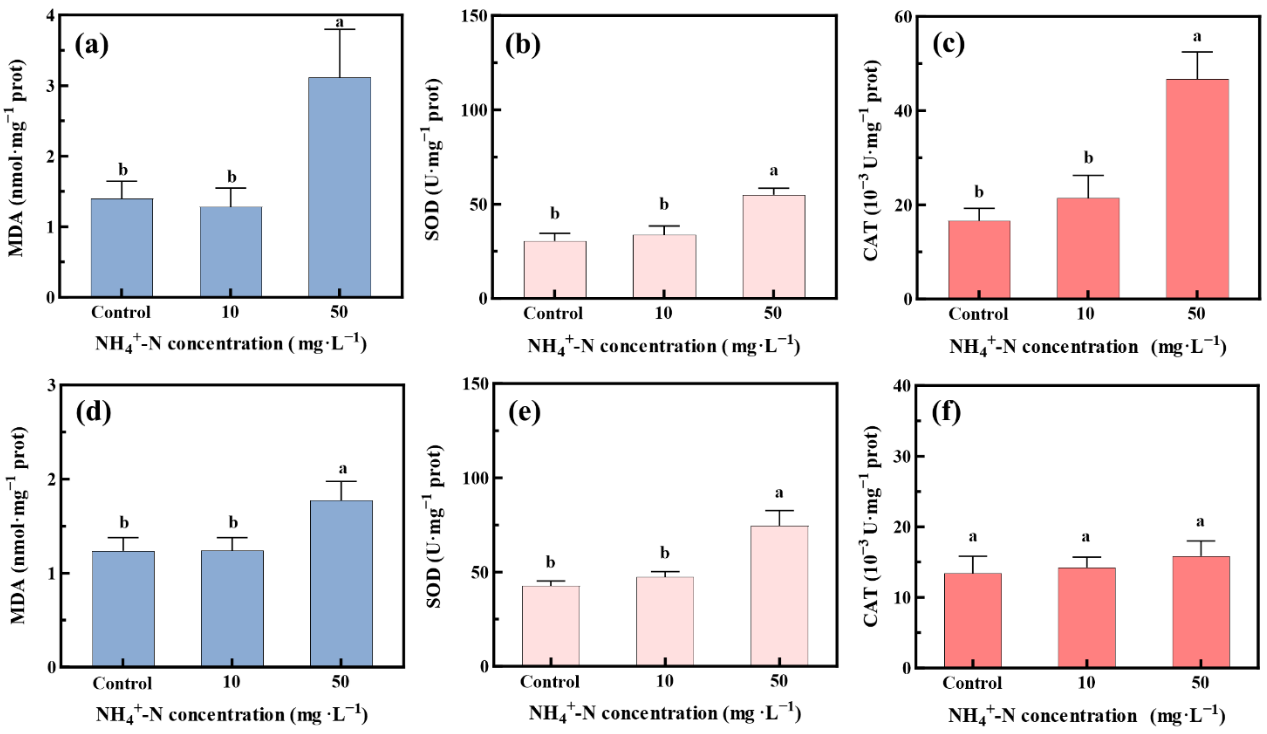

Figure S10. Oxidative stress status within *Oocystis lacustris* cells treated with different concentrations of NH_4^+-N on the 8th day - November 2022. (**a–c**) MDA concentration and SOD and CAT activities within algal cells in the EX phase; (**d–f**) MDA concentration and SOD and CAT activities within algal cells in the STA phase. (Different letters on adjacent bars indicate significant differences ($p < 0.05$), while the same letter indicates no significant difference.)

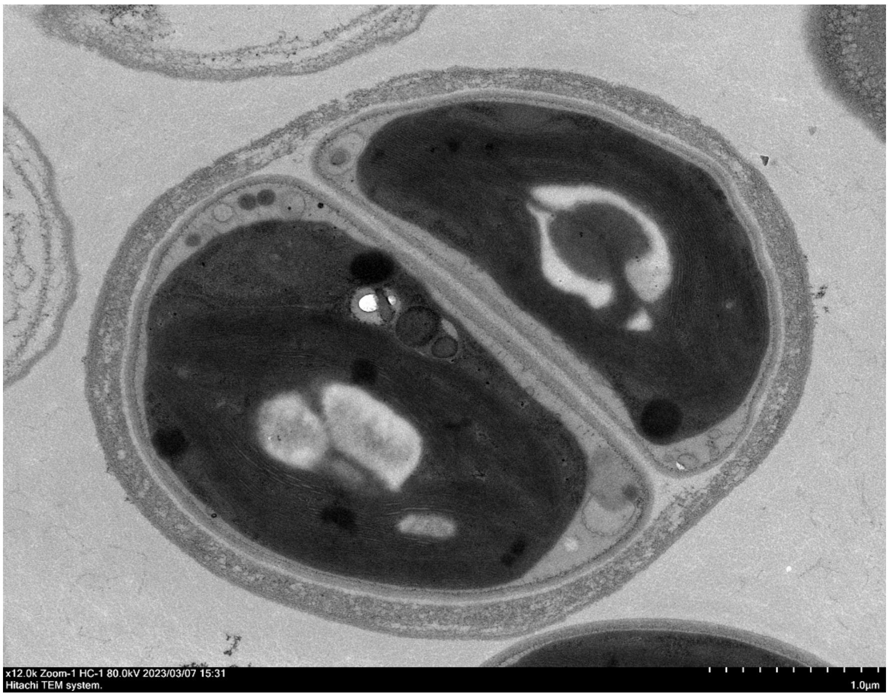

Figure S11. Microscopic morphology of *Oocystis lacustris* cells in the EX phase on the 8th day: Two cells enveloped in a pectinaceous gelatinous sheath of control group.

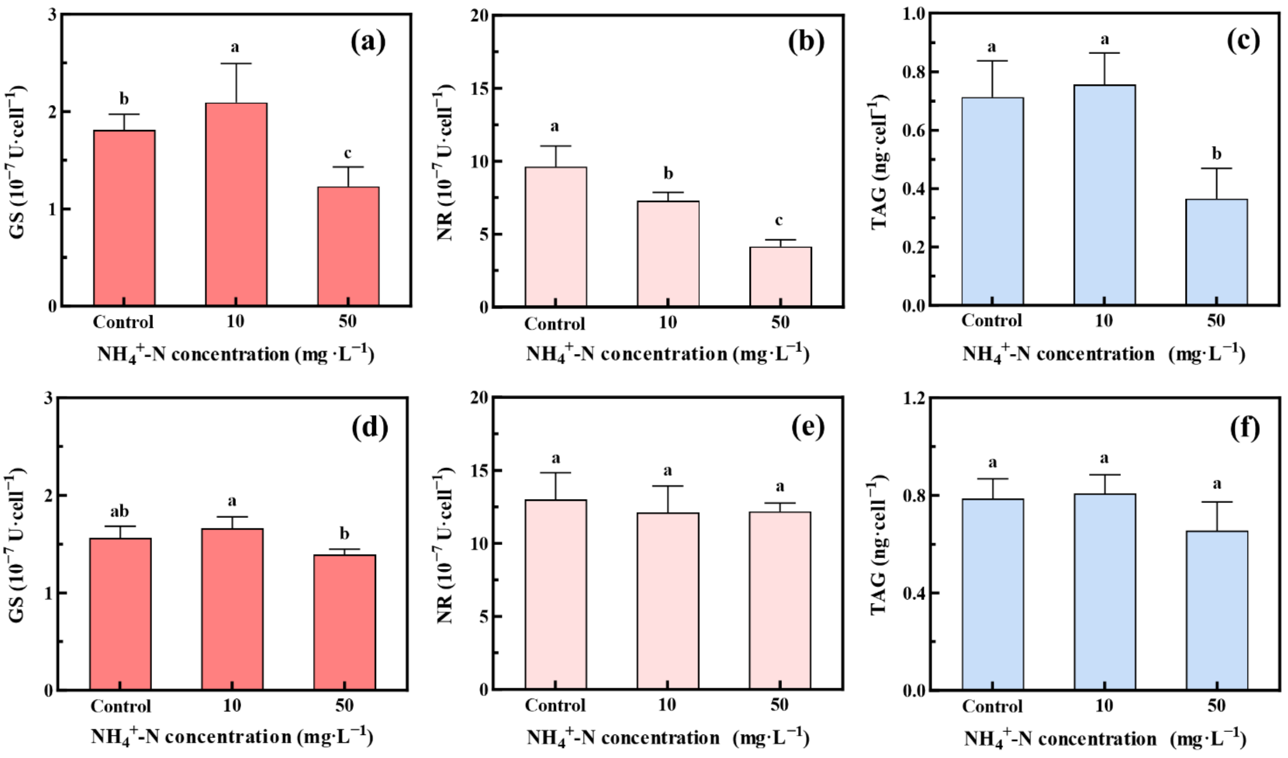

Figure S12. Changes in nitrogen metabolism enzymes and TAG activity within *Oocystis lacustris* treated by different NH_4^+-N concentrations - September 2022: (**a**) GS, (**b**) NR, and (**c**) TAG within algal cells in the EX phase; (**d**) GS, (**e**) NR, and (**f**) TAG within algal cells in the STA phase. (Different letters on adjacent bars indicate significant differences ($p < 0.05$), while the same letter indicates no significant difference.)

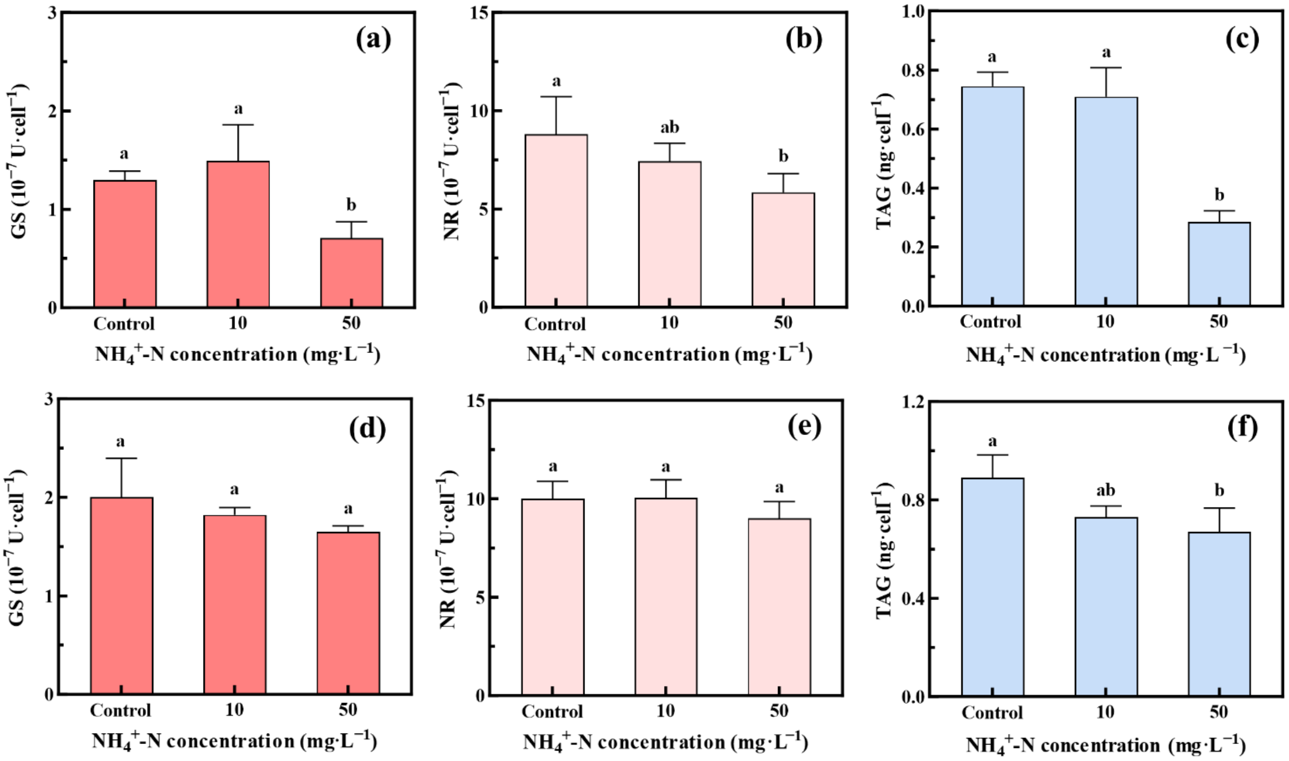

Figure S13. Changes in nitrogen metabolism enzymes and TAG activity within *Oocystis lacustris* treated by different NH_4^+-N concentrations - November 2022: (**a**) GS, (**b**) NR, and (**c**) TAG within algal cells in the EX phase; (**d**) GS, (**e**) NR, and (**f**) TAG within algal cells in the STA phase. (Different letters on adjacent bars indicate significant differences ($p < 0.05$), while the same letter indicates no significant difference.)
